# Staged Incentive and Punishment Mechanism for Mobile Crowd Sensing

**DOI:** 10.3390/s18072391

**Published:** 2018-07-23

**Authors:** Dan Tao, Shan Zhong, Hong Luo

**Affiliations:** 1 School of Electronic and Information Engineering, Beijing Jiaotong University, Beijing 100044, China; 15120043@bjtu.edu.cn; 2 Institute of Information Engineering, Chinese Academy of Sciences, Beijing 100093, China; 3 School of Computer Science, Beijing University of Posts and Telecommunications, Beijing 100876, China; luoh@bupt.edu.cn

**Keywords:** mobile crowd sensing, incentive mechanism, punishment mechanism, data utility, Stackelberg game, reputation accumulation

## Abstract

Having an incentive mechanism is crucial for the recruitment of mobile users to participate in a sensing task and to ensure that participants provide high-quality sensing data. In this paper, we investigate a staged incentive and punishment mechanism for mobile crowd sensing. We first divide the incentive process into two stages: the recruiting stage and the sensing stage. In the recruiting stage, we introduce the payment incentive coefficient and design a Stackelberg-based game method. The participants can be recruited via game interaction. In the sensing stage, we propose a sensing data utility algorithm in the interaction. After the sensing task, the winners can be filtered out using data utility, which is affected by time–space correlation. In particular, the participants’ reputation accumulation can be carried out based on data utility, and a punishment mechanism is presented to reduce the waste of payment costs caused by malicious participants. Finally, we conduct an extensive study of our solution based on realistic data. Extensive experiments show that compared to the existing positive auction incentive mechanism (PAIM) and reverse auction incentive mechanism (RAIM), our proposed staged incentive mechanism (SIM) can effectively extend the incentive behavior from the recruiting stage to the sensing stage. It not only achieves being a real-time incentive in both the recruiting and sensing stages but also improves the utility of sensing data.

## 1. Introduction

Nowadays, various human-carried mobile devices (e.g., smartphones and wearable devices) are ubiquitous and widely used iand have rich sensors are built-in and multiple radios provided [1]. This trend enables individuals with mobile devices to sense, collect, process, and distribute data around people at any time and place. By combining “crowdsourcing” with a distributed problem-solving model, “mobile sensing” offers a novel sensing paradigm with greater potential, to leverage a large contributing crowd to perform sensing tasks at a larger scale [2]. This novel sensing paradigm is often called mobile crowd sensing (MCS), and it has been leveraged to develop various applications, such as environmental monitoring [3], indoor localization [4,5,6], and social networking [7], etc.

In mobile crowd sensing, the sensing platform releases a sensing task to mobile users through the network. Mobile users who are willing to participate in sensing tasks accept the sensing task and collect and upload sensing data to the sensing platform. During this process, the following problems may occur: Firstly, the sensing platform may not recruit enough mobile users to participate in the sensing task. Secondly, mobile users who are willing to join in a sensing task may not be kept for long-term participation and maintain a positive state. In these cases, the quality and reliability of sensing data cannot be guaranteed during the data-collecting process. In crowdsensing, appropriate rewards are always expected to compensate the participants for their consumption of physical resources, involvement in manual efforts, and privacy disclosure [8].

Meanwhile, the sensing platform in the ubiquitous MCS network has a weaker binding on mobile users compared to that in the P2P network. The distinct characteristics of mobiles users mentioned above, will result in unqualified participants or even malicious behaviors, such as updating fake data or low-quality data, and so on.

Recently, some researchers have done pioneer works on motivating users to contribute their resources. Most of the existing incentive mechanism studies can be divided into two categories. One is to consider the influence of payment on the crowd to attempt to increase the number of participants. However, payment has been determined before the task is completed, regardless of the participants’ sensing behaviors being stimulated in the sensing stage. The other is to consider individual data and try to motivate the participants through a utility calculation after the completion of sensing task. However, these solutions overlook the effect of a crowd’s spatial-temporal distribution and data utility on payment cost. In addition, very few studies on punishment mechanism have been proposed in the context of MCS.

Motivated by this, this paper explores a staged incentive and punishment mechanism. The main contributions of our works are summarized as follows.

- First, we propose a staged incentive mechanism to extend the incentive process from the recruiting stage to the sensing stage, and establish a framework of staged incentive and punishment mechanisms for Mobile Crowd Sensing.
- Second, we introduce the payment incentive coefficient and design a Stackelberg-based game method in the recruiting stage. The game interaction is utilized to recruit participants in order to enhance the participants’ motivation to join in a sensing task.
- Third, in the sensing stage, we propose a sensing data utility algorithm for the interaction. The data utility affected by time–space correlation is used to filter out the winners after the sensing task to improve the quality of the sensing data.
- Finally, a reputation accumulation-based punishment mechanism is designed to introduce binding on malicious participants to save costs and lower resource waste.

The remainder of this paper is organized as follows. Section 2 overviews the related work. Section 3 gives the problem description. In Section 4 and Section 5, a staged incentive and punishment mechanism is proposed. A simulation and numerical results are given in Section 6, and we conclude this paper in Section 7.

## 2. Related Work

### 2.1. Incentive Mechanism

At present, most studies of incentive mechanisms in MCS have focused on the methods themselves, which can be classified into three categories: entertainment & game incentives, reward incentives, and social relation & service incentives [9]. Different methods may bring different incentive effects. It has been pointed out that reward incentive mechanisms tends to be the better ones.

For reward incentives, it is crucial for MCS that qualified participants are chosen and effective reward incentives are conducted based on behaviors according to the demands of sensing tasks. Existing studies on incentive mechanisms are mainly based on the idea of game theory. Specifically, the sensing platform assigns a task, and the platform and mobile users (potential participants) play a dynamic game to select participants and determine the payment price. There are two kinds of game ideas: positive auction and reverse auction. Some of the representative studies are discussed in the following text.

Lee et al. [10] first introduced an economic model for user participation and proposed a reverse auction-based dynamic price incentive mechanism. They removed the burden of accurate pricing for user sensing data and adapted it to dynamic changes in the user’s true valuation. However, the solution did not consider whether data collected was valid or if pricing was reasonable. Shah-Mansouri et al. [11] proposed a Profit Maximizing Truthful auction mechanism for mobile crowdsourcing systems, which aimed to maximize the profit of the platform while providing satisfying rewards to the smartphone users. Yang et al. [3,12] considered two system models from two different perspectives: the crowdsourcer-centric model, where the crowdsourcer provided a fixed reward to participating users, and the user-centric model, where users could have reserve prices for the sensing service. Nan et al. [13] proposed the Cross-Space, multi-Interaction-based dynamic Incentive mechanism (CSII), which estimated the value of a task based on the sensing context and historical data. It then had multiple interactions with both the task requester and potential contributors to adjust the budget and select suitable people to form the worker group.

Zhao et al. [14] investigated the frugal online incentive problem based on an online auction model, where users reported their strategic profiles to the crowdsourcer in an online mode, and the crowdsourcer selected users before a deadline to complete a specific number of tasks while minimizing the total payment. They designed two kinds of online mechanisms, namely, Frugal-OMZ and Frugal-OMG. Zhu et al. [15] proposed an incentive mechanism by combining reverse auctions and Vickrey auctions. The incentive mechanism effectively improved the fairness of bids without considering the problem of crowd imbalance. Considering that limited research efforts were made to address the quality of the recruited crowd, Wang et al. [16] presented an auction formulation for quality-aware and fine-grained MCS which minimized the expected expenditure subject to the quality requirement of each subtask.

Chakeri et al. [17] developed an incentive mechanism for crowd sensing markets with imperfect information. They presented an iterative game framework where the equilibrium of the market was achieved after a number of iterations. Further, Chakeri et al. [18] proposed an incentive mechanism for crowd sensing with multiple crowd sourcers. They considered two different pricing mechanisms—the crowd sourcers fixed the rewards in advance, or the crowd sourcers dynamically set the rewards in order to maximize their own utilities. To answer the challenges of participant recruitment in continuous sensing, Azzam et al. [19] proposed a recruitment system based on the stability of the spatio-temporal availability of participants in the AoI over the specified time period. Unlike previous works, Guo et al. [20] assumed that each sensing task could be performed by more than one users, but its single profit was invariable. Additionally, the sensing tasks that each mobile user could deal with were determined, which made the fees charged by each user be determined. They first proved the NP hardness of this problem, and then adopted a modified greedy algorithm, called gPUR, to solve it.

To sum up, the existing solutions cannot fully consider the impacts on incentive from different factors at different stages. Motivated by this, in this paper, we propose a staged incentive mechanism, which fully considers double effects, namely, (i) the effect of a crowd’s spatial-temporal distribution on the payment cost, and (ii) the effect of participants’ behaviors on data utility.

### 2.2. Punishment Mechanism

The punishment mechanism is widely used in the P2P network, which is a kind of management and constraint mechanism for the nodes’ behaviors in the network [21]. In such a distributed network environment, because of the mistrust among nodes and the selfishness of nodes, free riding, joint fraud, and arbitrary termination of services occur frequently, which can seriously affect the overall system efficiency [22]. Researchers have tried to introduce punishment mechanisms into the P2P network to constrain the nodes’ behavior.

Researchers have proposed some inclusive, dynamic punishment algorithms. For example, Zhang et al. [23] designed a trust-based punishment algorithm to suppress the nodes’ malicious behavior in the network. Zhang et al. [24] put forward an optimal punishment mechanism based on the nodes’ selfish behavior to achieve the whole benefit of data transmission. Wen et al. [25] designed a static and dynamic punishment mechanism by combining the Tat strategy and game theory. It avoided the inadequacy in the distinctiveness of the Tat strategy by setting the punishment coefficient. At present, there have been few studies involving punishment mechanisms in MCS. Studies have focused on how to establish a reputation model to reduce participants’ malicious behavior. Nan et al. [13] set up a fuzzy reputation model to accumulate a participant’s reputation. Participants with high reputation values had a greater chance of compensation.

In conclusion, the existing incentive mechanisms adopted the reputation model to encourage and compensate high-quality participants but without considering the sensing platform’s binding force on participants. In this paper, we introduce data utility to accumulate reputations for participants, and malicious participants are filtered out by setting a certain reputation threshold.

## 3. Problem Description

### 3.1. Sensing Task Model

**Figure 1:**
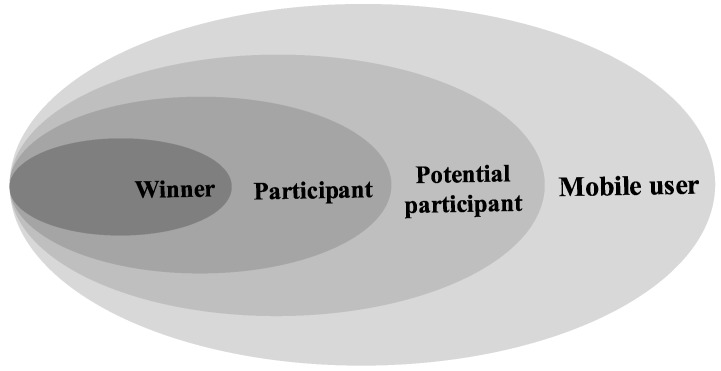
Group relationship.

Considering that a sensing task is complex and diverse in the MCS network, we first model a sensing task and give some definitions here. The group relationship is given in Figure 1.

- Sensing platform: This is the core and is responsible for releasing sensing tasks and choosing participants.
- Potential participant: Mobile users who have the possibility of participating in a sensing task.
- Participant: Potential participants who accept and participate in a sensing task.
- Winner: Participants who complete a sensing task and finally, get rewards.

In this paper, a complex sensing task is decomposed into multiple simple subtasks in spatio-temporal dimensions. Generally, a simple sensing task can be represented as a six-tuple:

(1) task=<site,radii,gt,lt,content,cost>,

where site is the location information specified by task, and radii is the radius of a circular sensing region with the center of a task site. gt and lt denote a sensing task’s required time and expiry time, respectively. That is, the data collected by participants within [gt-lt,gt+lt] will be effective. content represents the content of a sensing task. cost is the total rewards paid.

**Figure 2:**
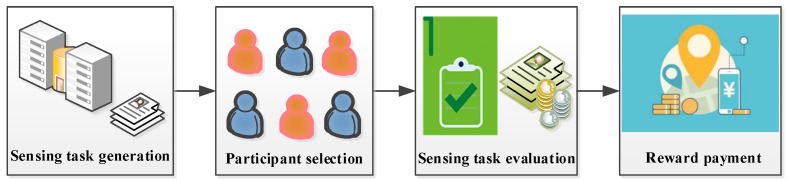
The incentive process of mobile crowd sensing (MCS).

The incentive process of MCS can be divided into four phases: sensing task generation, participant selection, sensing task evaluation, and reward payment, as illustrated in Figure 2. Through a comprehensive analysis, the sensing platform releases a specific sensing task and selects an appropriate participant set from the cohort of mobile users. The data submitted by participants can be evaluated based on data utility by the sensing platform. Finally, the qualified participants determined to be winners get rewards, and the whole incentive process is complete.

### 3.2. Crowd Analysis

In short, mobile crowd sensing is a new kind of sensing paradigm which is to collect data with the involvement of mobile users. For a sensing task, the difficulty in recruiting enough participants from different scales of crowd are different. Intuitively, the smaller the crowd scale is, the greater the difficulty of recruiting participants will be, and thus, the higher the incentive cost required. In general, the distribution of crowd in a city follows a certain regularity. So, it is crucial for participant recruitment to fully understand the regularity of crowd distribution.

**Figure 3:**
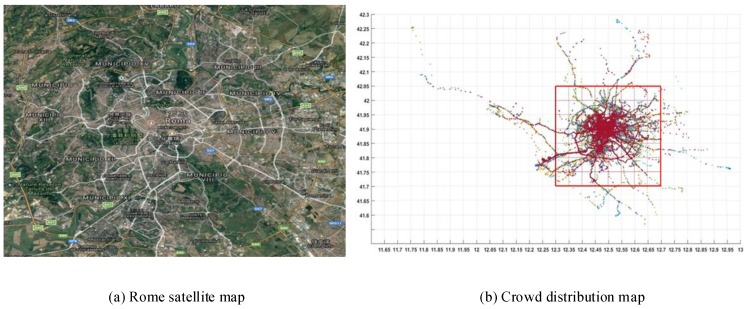
Maps.

In this paper, the realistic crowd distribution in Rome city from the website http://www.crawdad.org is used. Figure 3a gives the satellite map of Rome, and Figure 3b shows the positions where pedestrians have been in a time period of 30 days. It can be seen that the crowd distribution is mainly concentrated in the urban area marked with red grid; only a very small part of crowd have appeared in the remote area.

**Figure 4:**
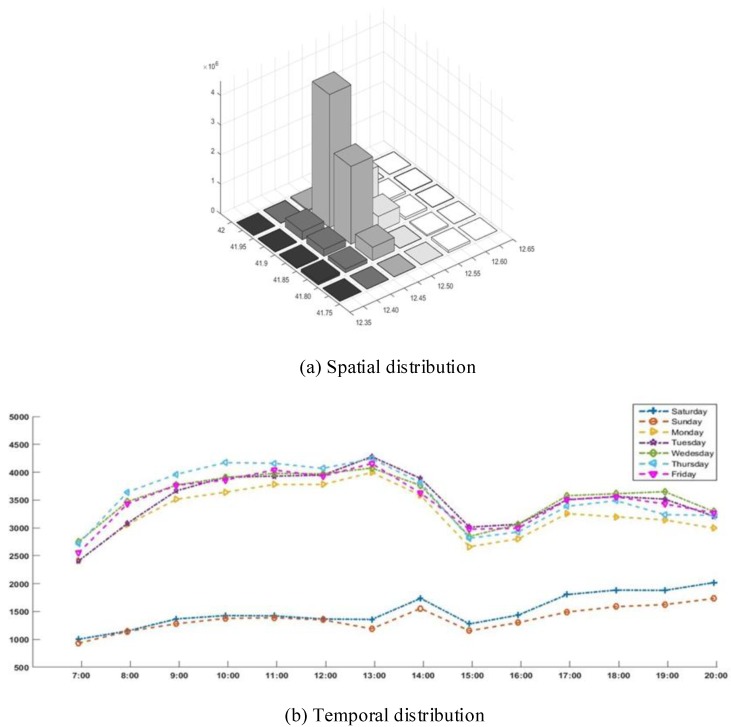
Spatial and temporal distribution of the crowd.

In the active region, the longitude is in the range of 12.3 to 12.7, and the latitude is in the range of 41.7 to 42. Through meshing, this region can be divided into several subregions. As shown in Figure 4a, the occurrence numbers of pedestrians in all the grids (sub-regions) were counted. Then, the crowd distribution was analyzed from the temporal dimension. Here, one week was considered to be a completed statistical cycle. The average number of pedestrians’ GPS data obtained from 7 a.m. to 8 p.m. each day in 30 days was counted in order to reflect the trend of pedestrian flow in different periods. The temporal distribution of pedestrians in 30 days is illustrated in Figure 4b.

From the data shown in Figure 4, we observe that the crowd distribution is unbalanced. This kind of node distribution will affect the coverage quality of mobile crowd sensing, which is apparently different from the node distribution in mobile ad-hoc and sensor networks [26,27,28].

- **Imbalance of temporal distribution**: The size of the crowd varies in different time periods, increasing, evidently, in the peak period and relatively reducing in the off-peak period.
- **Imbalance of spatial distribution**: The densities of the crowds in different regions are significantly different. The size of the crowd in hot areas is much larger than that in other areas of the city.

In MCS, mobile users are the sellers of sensing data, and the sensing platform is the purchaser of sensing data. Mobile users are sensitive to the rewards paid, which will directly impact the participation of the crowd. Here, the reward can be set as the product of the payment coefficient and data utility. The payment coefficient is flexibly set to regulate the rewards of sensing activity, thus motivating the enthusiasm of participants. Obviously, the number of participants is in proportion to the payment coefficient based on the size of the crowd.

## 4. Staged Incentive Mechanism

### 4.1. Staged Incentive Mechanism Framework

**Figure 5:**
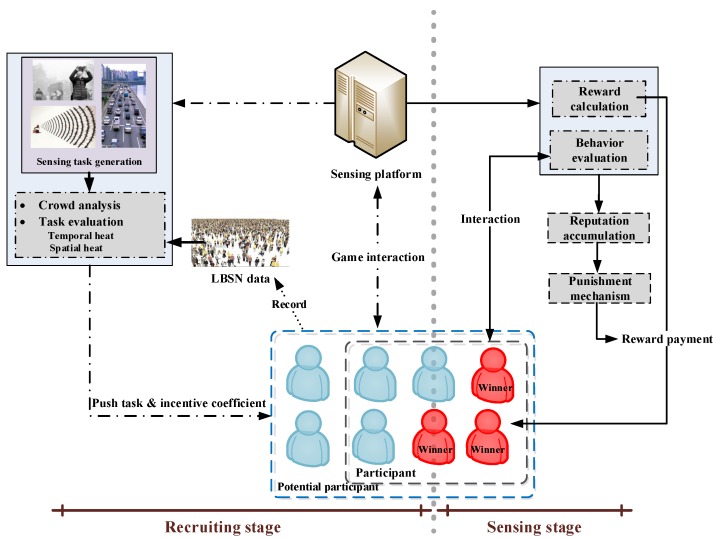
Staged incentive mechanism framework.

A staged incentive mechanism framework is shown in Figure 5, which contain two stages: the recruiting stage and the sensing stage. Note that, the punishment mechanism is involved at the end of sensing stage which is detailed in Section 5.

- **Recruiting stage**: The sensing platform assesses the sensing tasks by analyzing the mobile crowd using location-based social network (LBSN) data. Based on the game model, the participant set is achieved to solve the problem of insufficient participants caused by the imbalance in the mobile crowd distribution.
- **Sensing stage**: The sensing platform evaluates the behaviors of participants and calculates the data utility to guide participants to collect data at the optimal time and location. Meanwhile, the participants can be chosen with reference to their reputation accumulation in order to inhibit the participation of malicious participants.

### 4.2. Recruiting Stage

In this stage, the sensing platform is mainly responsible for recruiting participants from the pool of potential participants. Generally, the sensing platform and each potential participant determine a certain reward in advance through an auction and game in order to attract potential participants to participate in the sensing task. However, it is hard to generate a reasonable payment without referring to the quality of sensing data from potential participants before the end of the sensing task. Moreover, changes in the sensing time, gt, and sensing location, site, will bring changes in the number of potential participants, which makes the difficulty of recruiting participants variable.

Motivated by this, we used LBSN data to assess a sensing task from spatio-temporal dimensions and proposed a payment incentive coefficient-based game interaction solution.

#### 4.2.1. Payment Incentive Coefficient Calculation

In our solution, the reward for a participant, reward, is calculated from the payment incentive coefficient, *C*, and the data utility, Utility, as follows:

(2) reward=C×Utility.

Formula (2) reveals that the larger the payment incentive coefficient is, the more the participants will be paid under the same Utility. *C* can be calculated from a sensing task’s temporal heat and spatial heat (definitions are given in the following subsection), and reflects the number of potential participants in a given region. Specifically, the lower the number of potential participants is, the larger *C* is, and the greater the difficulty of recruitment will be.

A sensing task is generally a multiperiod, multisite task. The distribution of the number of potential participants in different periods and at different task sites will be imbalanced. Apparently, a unified incentive mechanism is not conducive to participant recruitment. Therefore, we decomposed a multiperiod, multitask site sensing task into a series of simple subtasks to form a task set, and each simple sub-task was evaluated based on LBSN data to directly impact the size of the crowd and the level of activity. The parameters used are summarized in Table 1.

It is assumed that a sensing task released contains *n* periods and *m* task sites (in our work, 14 sensing tasks (from 7 a.m. to 8 p.m.) were released per hour per day at 4 task sites, so *n* = 14 and *m* = 4.). A period set can be denoted as *T* = $\{T_{1}$, $T_{2}$, ..., $T_{n}\}$, where $T_{i}$ is a time period. A task site set can be denoted as Θ, Θ = $\{s_{1}$, $s_{2}$, ..., $s_{m}\}$, where $s_{j}$ is a task site. So, the task set can be represented as $task_{T,\Theta }$, where $task_{T_{i},s_{j}}$ is a subtask in the $s_{j}$ region during $T_{i}$. In this way, a large-scale sensing task can be decomposed into multiple simple subtasks.

To facilitate the evaluation of subtasks, the mobile users are represented during $T_{i}$ by

(3) $$U_{T_{i},\Theta }=U_{T_{i},s_{1}}\cup U_{T_{i},s_{2}}\cup ...\cup U_{T_{i},s_{m}},$$

where $U_{T_{i},\Theta }$ is the universal set of mobile users (Note that, throughout the rest of the paper, unless otherwise mentioned, “potential participant” refers to “mobile user”.) during $T_{i}$; $U_{T_{i},s_{j}}$ is the set which consists of the mobile users who check into the $s_{j}$ region during $T_{i}$.

**Temporal heat** is one critical evaluation indicator. The number of check-ins is directly proportional to the total number of mobile users during a certain period. So, the ratio of the number of check-ins during a certain period to the total number of check-ins during all periods can be denoted as temporal heat.

The temporal heat of $task_{T_{i},\Theta }$ can be represented by $heat_{T_{i}}$.

(4) $$heat_{T_{i},\Theta }=\frac{\sum_{u\in U_{T_{i},\Theta }}R_{T_{i},\Theta }\left(u\right)}{\sum_{u\in U_{T,\Theta }}R_{T,\Theta }\left(u\right)},$$

where $R_{T_{i},\Theta }\left(u\right)$ denotes the number of check-ins during $T_{i}$ for user *u*. Similarly, $R_{T,\Theta }\left(u\right)$ denotes the total number of check-ins for user *u*.

**Spatial heat** is the other critical evaluation indicator, which can be denoted as the ratio of the number of mobile users in the $s_{j}$ region to the number of mobile users in the Θ region during a certain period, $T_{i}$. Here, the richness of mobile users can be measured by the information entropy, En(). A greater information entropy reflects more abundant mobile users. The spatial heat of $task_{T_{i},s_{j}}$ can be represented as $heat_{T_{i},s_{j}}$.

(5) $$heat_{T_{i},s_{j}}=\frac{\sum_{u\in U_{T_{i},s_{j}}}R_{T_{i},s_{j}}\left(u\right)}{\sum_{u\in U_{T_{i},\Theta }}R_{T_{i},\Theta }\left(u\right)}\times En\left(U_{T_{i},s_{j}}\right),$$

where $R_{T_{i},s_{j}}\left(u\right)$ denotes the number of check-ins in the $s_{j}$ region during $T_{i}$ for user *u*, and $R_{T_{i},\Theta }\left(u\right)$ denotes the total number of check-ins during $T_{i}$ for user *u*. $En(U_{T_{i},s_{j}})$ is the information entropy of mobile users in the $s_{j}$ region during $T_{i}$, which can be defined as follows:

(6) $$En\left(U_{T_{i},s_{j}}\right)=-\sum_{u\in U_{T_{i},s_{j}}}p_{T_{i},s_{j}}\left(u\right)\times \log p_{T_{i},s_{j}}\left(u\right),$$

where $p_{T_{i},s_{j}}\left(u\right)$ is denoted in Formula (7).

(7) $$p_{T_{i},s_{j}}\left(u\right)=\frac{R_{T_{i},s_{j}}\left(u\right)}{R_{T_{i},\Theta }\left(u\right)},u\in U_{T_{i},s_{j}}.$$

Further, the payment incentive coefficient, $C_{T_{i},s_{j}}$, for $task_{T_{i},s_{j}}$ is defined by considering the temporal heat and spatial heat together, which can be represented as follows. We conclude that the greater $heat_{T_{i},\Theta }$ and $heat_{T_{i},s_{j}}$ are, the easier the participant recruitment gets, and thus, the less payment incentive coefficient $C_{T_{i},s_{j}}$ becomes. In this way, we can spend less to achieve participant recruitment:

(8) $$C_{T_{i},s_{j}}=\frac{{\bar{heat}}_{T_{i},\Theta }}{heat_{T_{i},\Theta }}\times \frac{{\bar{heat}}_{T_{i},s_{j}}}{heat_{T_{i},s_{j}}},$$

where ${\bar{heat}}_{T_{i},\Theta }$ = $\sum_{i=1}^{n}heat_{T_{i},\Theta }$/*n* and ${\bar{heat}}_{T_{i},s_{j}}$ = $\sum_{j=1}^{m}heat_{T_{i},s_{j}}$/*m*.

#### 4.2.2. Strackelberg-Based Game Interaction

The Strackelberg game, as a kind of non-cooperative game, can be composed by two actors: the leader and the follower. Its main idea is that the leader first takes action, and then followers adjust their own strategies according to the leader to maximize their own benefits [29]. In the recruiting stage, the sensing platform is regarded as the leader; the potential participants are regarded as followers.

Based on the Strackelberg game, we figure out payment incentive coefficients by analyzing the activity information of crowd. Then, the sensing platform releases the payment incentive coefficients, and meanwhile, the potential participants as followers determine whether to upload the expected payment incentive coefficient and participate. Finally, the sensing platform selects participants based on the payment incentive coefficients uploaded by potential participants, and then, a collection of participants is formed.

Next, we discuss the game interaction process of the recruiting stage. Each potential participant, *u* = (ω,c), has two attributes, where ω = 0(1) denotes a potential participant will (will not) accept a sensing task and report his/her expected payment incentive coefficient, *c*.

After the game interaction, all potential participants who decide to participate in the sensing task form a collection of participants, $U^{\prime }$ = $\{u_{1}^{\prime }$,$u_{2}^{\prime }$,...,$u_{k}^{\prime }\}$. Similarly, each participant, $u^{\prime }$ = (ω,c), can also be defined with the meanings explained above.

Our solution is based on the following three assumptions:

- Each potential participant is rational. That is, a potential participant, *u*, can decide whether to report the expected payment incentive coefficient, *c*, or not according to the *C* issued by the sensing platform.
- In the game interaction process, each potential participant is independent and identically distributed. In other words, the expected payment incentive coefficient, *c*, reported by a potential participant has nothing to do with other participants.
- During the game interaction process, potential participants cannot communicate with each other. That is, $u_{i}$ knows nothing about any $c_{k}$ of $u_{k}$ where $u_{k}\in$ ($U-u_{i}$).

The Strackelberg-based game interaction algorithm (see Algorithm 1) can be described as follows.

**Algorithm 1** Strackelberg-based game interaction process
1:The sensing platform releases a sensing task, task, and calculates payment incentive coefficient, *C*, whose upper limit is defined as CTH;  2:The sensing platform pushes task and *C* to the set of potential participants *U*;  3:For each $u_{i}\left(\omega_{i},c_{i}\right)\in U$, he/she decides and reports ($\omega_{i},c_{i}$) to the sensing platform, and the sensing platform forms a collection of potential participants, $U=\{u_{1}\left(\omega_{1},c_{1}\right),u_{2}\left(\omega_{2},c_{2}\right),...\}$;  4:The sensing platform conducts statistics on *U*:4.1 if $\exists u_{i}\left(\omega_{i},c_{i}\right)\in U$ and $\omega_{i}$ = 0  then if C<CTH;   then C←C+ε; go to step 2;  else go to step 5;4.2 if $\forall u_{i}\left(\omega_{i},c_{i}\right)\in U$ and $\omega_{i}$ = 1  then go to step 5; 5:The sensing platform chooses participants according to $c_{i}$ of $u_{i}$ whose $\omega_{i}$ = 0 in *U* and constructs a collection of participants, $U^{\prime }$;  6:**return** Participant set, $U^{\prime }$.


The payment incentive coefficient, *C*, of each round of game interaction is constantly adjusted with the correction factor, ε. Here, the default value of ε was set as 0.05.

### 4.3. Sensing Stage

After the recruiting stage, the sensing platform obtains the participant set, $U^{\prime }$. However, the participants do not start to collect data. Intuitively, the utility of sensing data is closely related to the sensing time and location. The timeliness of sensing data indicates that, the more the sensing time approaches the required time, the higher the data utility will become. For the sensing distance, a similar conclusion is obtained. Moreover, orientation information is introduced to measure the distribution of participants in the region of site. To motivate participants to collect data with high utility, we fully consider space–time correlations, especially in regard to the orientation information. The platform establishes real-time communication with participants and figures out the space–time correlation coefficient so that it can prompt participants to adjust their sensing behaviors in the region of the task site to enhance data utility.

#### 4.3.1. Time Correlation

The time correlation can be determined from the relationship between the sensing time, *t*, and a sensing task’s required time, gt. If there are two types of sensing data, $data_{1}$ and $data_{2}$, whose sensing times are, respectively, $t_{1}$ and $t_{2}$, while meeting $|t_{1}-gt|<|t_{2}-gt|$ and $t_{1},t_{2}\in \left[gt-lt,gt+lt\right]$ (lt is the expiry time defined in Section 3.1) with the other conditions kept the same, the time correlation of $data_{1}$ is higher than that of $data_{2}$.

**Figure 6:**
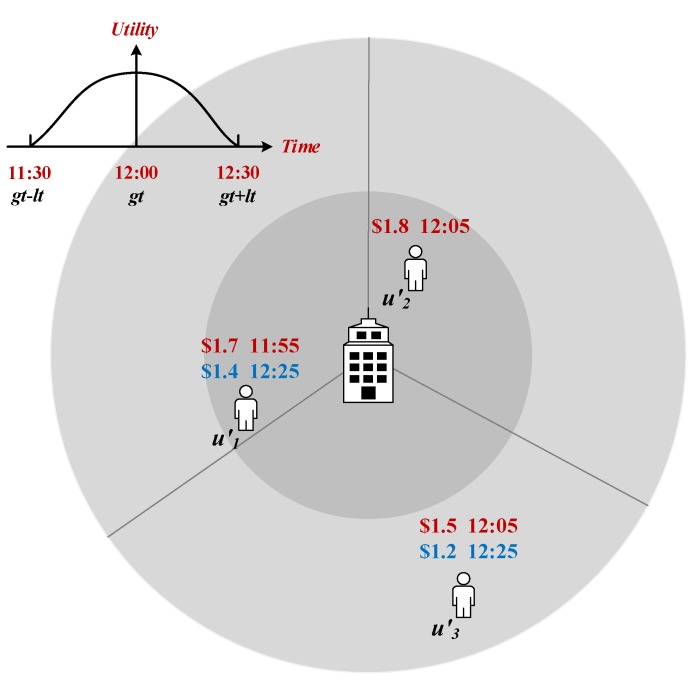
Data utility decided by different sensing times and distances.

In Figure 6, we show that the time correlation centers on gt and is attenuated on both sides. When t=gt, the time correlation reaches the maximum—1. For example, for participant $u_{1}^{\prime }$, from 11:30 a.m. to 12:30 p.m., the time correlation of data collected at 11:55 a.m. is higher than that collected at 12:25 p.m. So, the reward obtained at 11:55 a.m. ($1.7) is greater than that at 12:25 p.m. ($1.4) with other conditions being the same, generally. Based on the above analysis, we used Sigmoid and Sgn functions to describe the time correlation, VT(t), at the sensing time, *t*:

(9) VT(t)=2sgn(|t-gt|)×f(-|gt-t|)+sgn(-|t-gt|)

where *f*(*x*) = $\frac{1}{1+e^{-x}}$ and

$$sgn\left(x\right)=\left\{\begin{array}{ccc} 1 & , & x>0\\ 1/2 & , & x=0\\ 0 & , & x<0 \end{array}\right.$$

#### 4.3.2. Distance Correlation

The distance correlation can be determined by the distance, *d*, between the sensing location and the task site, *s*. If there are two types of sensing data, $data_{1}$ and $data_{2}$, whose sensing distances are, respectively, $d_{1}$ and $d_{2}$, while meeting $d_{1}<d_{2}$ and $d_{1},d_{2}\leq radii$ (radii is the radius of a circular sensing region with the center of a task site defined in Section 3.1) with all other conditions kept the same, the distance correlation of $data_{1}$ is greater than that of $data_{2}$.

Figure 6, shows that the distance correlation increases with a decrease in distance, *d*. When d=0, the distance correlation reaches the maximum—1.

For example, for participants $u_{2}^{\prime }$ and $u_{3}^{\prime }$, at the same time (12:05 p.m.), the distance correlation of data collected by $u_{2}^{\prime }$ is higher than that by $u_{3}^{\prime }$. So, the reward obtained by $u_{2}^{\prime }$ ($1.8) is larger than that by $u_{3}^{\prime }$ ($1.5) with other conditions being the same. Based on the above analysis, we also used Sigmoid and Sgn functions to describe the distance correlation, VD(d):

$$VD\left(d\right)=\left\{\begin{array}{ccc} 1 & , & d\leq \frac{radii}{\eta }\\ 2sgn(|d-\frac{radii}{\eta }|)\times f(-|d-\frac{radii}{\eta }\left|\right)+sgn\left(d-\frac{radii}{\eta }\right) & , & d>\frac{radii}{\eta }. \end{array}\right.$$

Here, considering that it is hard for a sensing location to completely coincide with a task site, we used η to define a small range [0, $\frac{radii}{\eta }$]. If $d\in [0,\frac{radii}{\eta }]$; its corresponding VD(*d*) can be valued as 1.

#### 4.3.3. Orientation Correlation

In the sensing process, the sensing data may come from different orientations in a circular region around the task site. Our solution fully considers the effects on data utility from the orientation information. A circular region can be equally divided into *s* sectors, which can be represented as $o_{1},o_{2},...,o_{s}$. Here, we set *s* = 3, which means a circular region can be equally divided into 3 subregions, each covering a sector of 120 degrees. Based on information theory, the information amount on each orientation can be calculated as *I*(*o*) = -log(*p*($o_{i}$)), where *p*($o_{i}$) denotes the proportion of participants in the orientation $o_{i}$. Considering that the range of values for the time correlation, VT(*t*), and the distance correlation, VD(*d*) is from 0 to 1, for convenient calculation, we normalized *I*(*o*) to get the orientation correlation, VO(*o*), as follows:

(10) $$VO\left(o\right)=\frac{I\left(o\right)-\min\{I\left(o_{1}\right),...,I\left(o_{s}\right)\}}{\max\left\{I\left(o_{1}\right),...,I\left(o_{s}\right)\right\}-\min\left\{I\left(o_{1}\right),...,I\left(o_{s}\right)\right\}}.$$

#### 4.3.4. Data Utility

A multidimensional sensing data can be depicted by data = {t,d,o,q}, where *q* (∈[0, 1.0]) denotes the quality of the sensing data itself, which can be affected by many kinds of objective factors but only the type of participants is used here. In our work, we classified participants into three types: high-quality, ordinary, and low-quality.

The utility of data reflects the value provided by a sensing task, which can be represented as follows:

(11) Utility=q×(αVT(t)+βVD(d)+γVO(o)),

where α, β, and γ are, respectively, the weights of the time correlation, VT(*t*), distance correlation, VD(*d*), and orientation correlation, VO(*o*). α+β+γ = 1, and their default values here are α=β=0.4 and γ=0.2.

The sensing platform can flexibly adjust these weights according to the needs of the sensing task. For example, for a time-sensitive sensing task, the weight of the time correlation, α, can be enhanced; similarly, for distance-sensitive sensing data, the sensing platform can add the weight of distance correlation, β.

In conclusion, in the sensing stage, the sensing platform and participants perform real-time interactions via intelligent information push & pull technology (IIPP). The sensing platform can obtain participants’ location information to calculate several space–time correlations, and then push them to the participants. The participants determine or adjust their own sensing behaviors according to the calculation results. Once the sensing data is uploaded, the sensing platform can calculate its data utility. Finally, the interaction process is finished.

## 5. Reputation Accumulation-Based Punishment Mechanism

Besides the proposed incentive mechanism, in this section, we introduce a reputation accumulation-based punishment mechanism to inhibit the negative impact from malicious participants. Here, a participant’s reputation can be used to determine whether this participant will be paid and if yes, how much payment he(she) should receive [30].

The reward for participant $u_{i}^{\prime }$ in a sensing task can be calculated by Formula (12):

(12) $$reward_{u_{i}^{\prime }}=c_{i}\times Utility_{u_{i}^{\prime }}.$$

The main difference between Formula (2) (in Section 4.2) and Formula (12) is that the payment incentive coefficient, *C*, can be substituted by $c_{i}$ which is participant $u_{i}^{\prime }$’s expected payment incentive coefficient, $c_{i}$, that is reported finally in the game interaction process.

**Figure 7:**
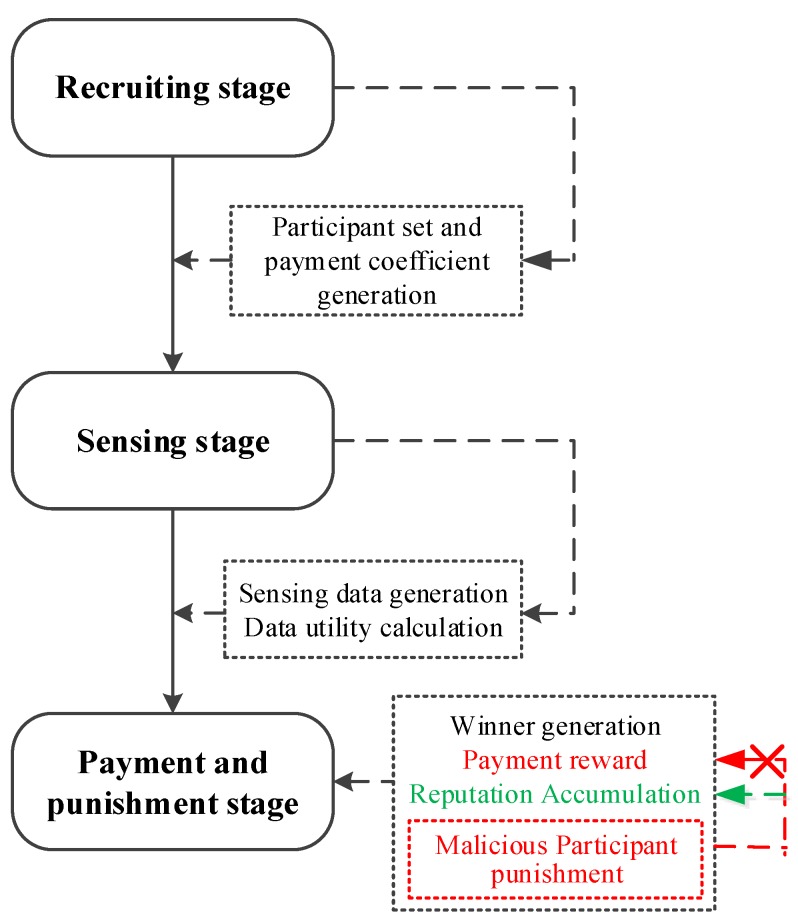
Flow of staged incentive and punishment mechanism.

The sensing platform screens the participants according to the rewards (i.e., payment cost) of the sensing task, $task_{T_{i},s_{j}}$. First, the participants can be sorted by data utility in descending order. Based on this, these participants can also be sorted by reward in ascending order. In this way, the sensing platform can pay less (rewards for participant) to get more winners who can provide a higher data utility. Subsequently, the sensing platform pays the corresponding rewards for the winners. The flow of the overall staged incentive and punishment mechanism is shown in Figure 7.

The basic idea of reputation accumulation is that the sensing platform specifies the data utility threshold, UTH, and the reputation deduction value, ζ. We regarded sensing data whose utility is less than UTH as malicious, and the reputation of a participant who reports malicious data is deducted by ζ. Otherwise, the reputation of participants who report good data accumulate as follows:

$$R_{u_{i}^{\prime }}=\left\{\begin{array}{ccc} \sum_{d=0}^{n}e^{-0.1d}\times Utility_{u_{i}^{\prime }}^{d} & , & Utility_{u_{i}^{\prime }}^{d}>UTH\\ \sum_{d=1}^{n}e^{-0.1d}\times Utility_{u_{i}^{\prime }}^{d}-\zeta & , & Utility_{u_{i}^{\prime }}^{d}\leq UTH, \end{array}\right.$$

where $R_{u_{i}^{\prime }}$ is the reputation of participant $u_{i}^{\prime }$. *d* denotes the times of sensing tasks that $u_{i}^{\prime }$ has participated in, where *d* = 0 means the current sensing task. For the reputation accumulation, the more recent the sensing behavior is, the greater the effect on the reputation of a participant will be. Hence, we introduce the attenuation factor, $e^{-0.1d}$, and the data utility reported by participant $u_{i}^{\prime }$ for the $d^{th}$ sensing task can be denoted by $Utility_{u_{i}^{\prime }}^{d}$. For example, $Utility_{u_{i}^{\prime }}^{0}$ is the data utility reported by participant $u_{i}^{\prime }$ for the current sensing task.

In this section, we design a reputation accumulation-based punishment mechanism. A malicious participant is defined as one whose reputation value is lower than the reputation threshold, RTH. As illustrated in Figure 7, the punishment mechanism has a major role in the determination of payment reward (marked with red), which sets strict criteria for the winners. In detail, at the end of a sensing task, the sensing platform firstly screens participants to achieve a winner set. Then, it does statistics on the reputations of winners. If a winner is a malicious participant (i.e., his reputation is less than RTH), the sensing platform only accumulates his reputation based on his data utility in this sensing task without paying reward to him. In other words, if a malicious participant is filtered out to be a winner, he can upload high-utility data to achieve continuous reputation accumulation, instead of getting a reward.

## 6. Simulation and Numerical Results

**Figure 8:**
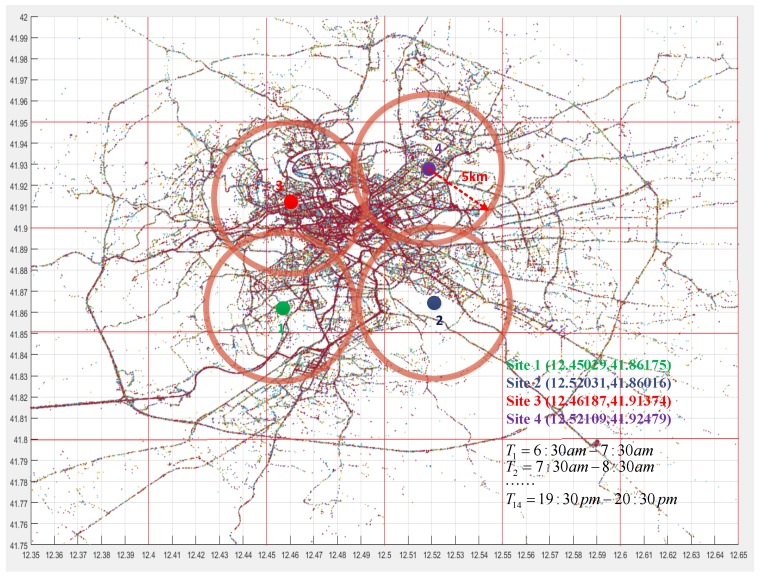
Some simulation parameters used.

Here, the simulation data came from the website (http://www.crawdad.org). These data include 21,817,851 location points of 360 pedestrians within a time period of 30 days. Each location point consists of a user’s ID, latitude and longitude, and time. Some simulation parameters used in our work are illustrated in Figure 8. There are four task sites in the monitored area, and their corresponding longitude and latitude data are marked. We drew a circle around each task site (a 5 km radius). For each circular area, we conducted 14 sensing tasks for each hour (from 7 a.m. to 8 p.m.) in one day, and 1,074,427 valid location points were obtained by uniform time sampling in the active area. Here, the cost ceiling of each sensing task was set as $15.

According to a report on mobile network users’ behaviors that was issued by CNNIC (http://www.cnnic.net.cn/), for different types of participants—high-quality, ordinary and low-quality ones—the sensory data quality is different. The value range of sensory data quality is [0, 1.0]. The higher the quality of a user is, the greater the sensory data quality he/she can provide will be. Without loss in generality, the simulation parameters of crowd can be set as follows. In the simulation, the percentages of high-quality, ordinary and low-quality users in 360 pedestrians were 20%, 60% and 20%, respectively. In addition, their corresponding participation probability intervals were [0.8, 1.0], [0.5, 1.0] and [0.2, 0.8], respectively.

We performed the simulation by using MATLAB tools. Particularly, we explored some primary parameters: the total data amount collected, the data utility, the number of winners, and the reputation accumulation, and thus, evaluated the performance of the positive auction incentive mechanism (PAIM), the reverse auction incentive mechanism (RAIM), and our proposed staged incentive mechanism (SIM) via extensive simulations. Each result shown here is the statistical average of 20 simulations.

### 6.1. Total Amount of Data Collected

To reduce the impact of users’ scale change, in this paper, we selected and analyzed data collected from 1624 sensing tasks in the simulation (For each task site, 14 sensing tasks (from 7 a.m. to 8 p.m.) were released in one day. Hence, for 4 task sites, there were, in total, 1624 sensing tasks issued within 30 days (about one month)). For different task sites, the cost ceilings of each sensing task were the same ($15).

Table 2 shows the total amount of data collected from four task sites in 30 days based on three kinds of incentive models. From the data shown in this table, the crowd distribution among four task sites was very unbalanced. For the reverse auction incentive mechanism (**RAIM**), the sensing platform does not announce the expected price in advance and only relies on the potential participants’ own decision-making, so the number of participants was less than that of other two algorithms. In addition, because the number of participants recruited by RAIM basically follows the crowd distribution, it was unable to effectively improve the numbers of participants at site 1 and site 2, and it also caused increased numbers of participants at site 3 and site 4. The positive auction incentive mechanism (**PAIM**) overcame the defect of RAIM by early release of price based on the sensing task assessment, which increased the overall number of participants. Particularly, PAIM motivated the enthusiasm of potential participants at different task sites to achieve a more balanced crowd distribution compared to RAIM. The proposed staged incentive mechanism (**SIM**) improved the overall number of participants while balancing the number of participants at the four task sites through the combined effect of the payment incentive coefficient and game theory-based interaction.

### 6.2. Data Utility

#### 6.2.1. Data Quality Distribution

**Figure 9:**
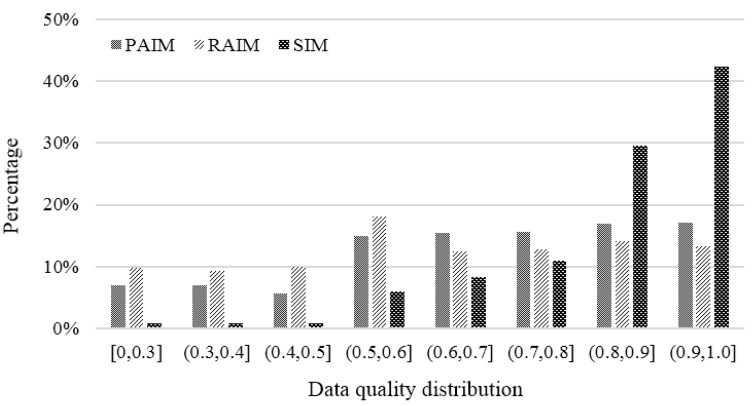
Comparison of data quality distribution.

We quantified the data quality within the range [0, 1.0]. It was concluded that the incentive coefficient has a great influence on the users’ participation enthusiasm. During peak periods, the more the number of potential participants in hotspots is, the less the payment incentive coefficient values are, and thus, the lower the enthusiasm of participants, especially low-quality ones, is. From the data in Figure 9, we can see that the data quality distribution of SIM was obviously superior to PAIM and RAIM. Specifically, the proportion of high quality data (0.8, 1.0) was 71.94% for SIM, which is about twice as high as the other two. This indicates that more payment costs are used to collect high-quality data in SIM, so that the whole payment cost can be reasonably employed during the incentive process. For PAIM and RAIM, the data quality distributions are relatively random, and a considerable portion of payment costs are used to pay for low-quality data.

#### 6.2.2. Data Delay Distribution

**Figure 10:**
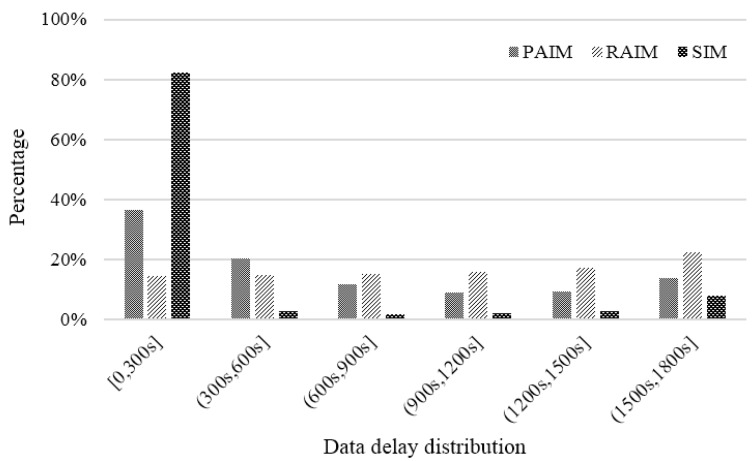
Comparison of data delay distribution.

Delay can be denoted as the collection time difference between the specified time (e.g., 12 p.m.) and the actual time (e.g., 11:55 a.m.; 12:05 p.m.). This actual collection time has a great impact on the utility of data because our incentive is to make more participants collect data close to the specified time to increase the reliability of collected data. As shown in Figure 10, the data delay distributions of PAIM and RAIM were relatively uniform, and complied with the principle of random sampling. However, for SIM, the proportion of low delay interval [0, 300], (300, 600] was 85.26%, which is, respectively, 1.5 times and twice as high as that of PAIM and RAIM. This means that SIM can simulate participants to collect data at a certain time using the time correlation coefficient.

#### 6.2.3. Data Distance Distribution

**Figure 11:**
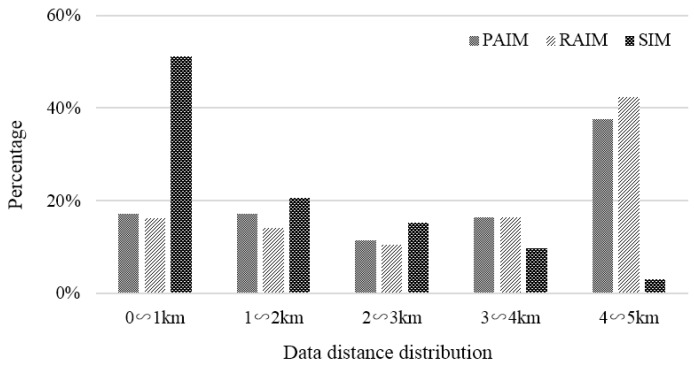
Comparison of data distance distribution.

Distance is denoted as the distance difference between the sampling location and the task point. The utility of data decreases with an increase in distance. Our incentive was to make more participants collect data close to the task point to improve the data reliability. From the statistical data in Figure 11, it can be seen that the amount of data collected by PAIM and RAIM was evenly distributed from 0 to 4 km, but the amount of data collected in the distance interval of 4–5 km was relatively high. This is because the distance factor is not considered in these two incentive models, so edge collection, which means that a lot of participants who enter or leave a given task area tend to participate in sensing tasks regardless of the influence of distance on data utility, inevitably happens. For SIM, the proportion of data collected within 0–1 km approached 51.06%, nearly three times that of PAIM and RAIM. This reveals that SIM can simulate participants to collect data at nearby task points based on the distance correlation coefficient.

#### 6.2.4. Data Orientation Distribution

In the simulation, the region of a task site was divided into three subregions, each covering a sector of 120 degrees. The data collection locations located in different orientations have different impacts on the data utility. We hoped that the collected data from different orientations would reflect different samples.

**Figure 12:**
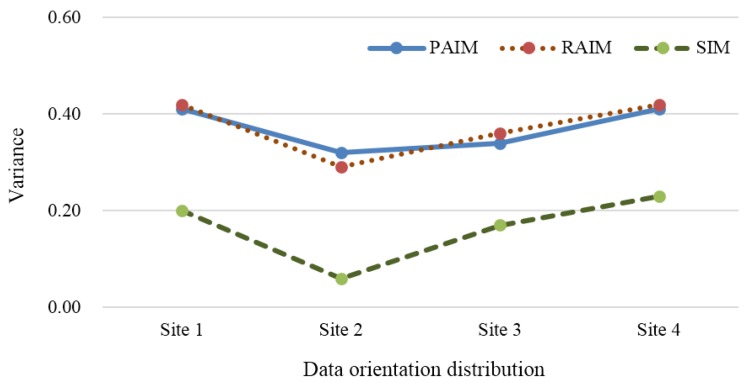
Comparison of data orientation distribution.

The variance in the amount of sensing data from different orientations for four task sites using PAIM, RAIM, and SIM are shown in Figure 12. We can see that the variance in the sensing data amount with SIM was less than that with PAIM and RAIM. Obviously, the value changed between 0.06 and 0.23. This is mainly because the orientation distributions of data collected for each task site by PAIM and RAIM meet a natural distribution, so most of the sensing data comes from one orientation which results in a great variance value. Our proposed SIM can effectively motivate the participants who are located in the direction with lower crowd distribution to join in the sensing task in order to make the data source distribution more balanced and the sensing data content richer.

#### 6.2.5. Brief Summary

**Figure 13:**
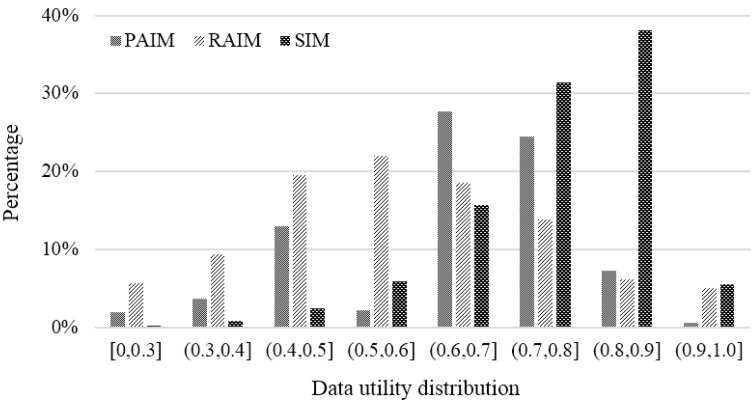
Comparison of data utility distribution.

In conclusion, according to Formula (10), the value range of the data utility was [0, 1]. In the process of motivation, we expect to obtain higher data utility by paying more payments to the participants who provide data with high utility. From the data in Figure 13, we can see that the data utility distributions of PAIM and RAIM basically obeyed a Gaussian distribution, and lots of data utilities were mainly distributed within the median utility interval from (0.4, 0.5] to (0.7, 0.8]. However, the curve of the data utility distribution of SIM obviously moved to high data utility. In detail, the data utility in these three utility intervals (0.6, 0.7], (0.7, 0.8] and (0.8, 0.9] was about 82.95% of the total, and it was about twice as much as those of PAIM and RAIM. In comparison, we found that SIM significantly improved the participants’ collection behaviors and promoted the total data utility under the same payment cost.

### 6.3. The Number of Winners and Reputation Accumulation

**Figure 14:**
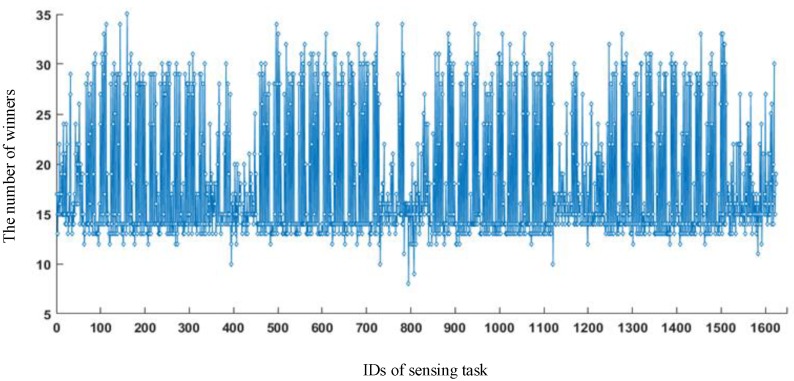
The number of winners for all sensing tasks.

In the simulation, the cost ceiling of a sensing task was set as $15. The number of winners filtered out for each sensing task is given in Figure 14. The horizontal axis shows the IDs of the sensing task. We found that the number of winners filtered out fluctuated within a range of 9–35. The lower the number of winners was, the greater the reward each winner obtained.

**Figure 15:**
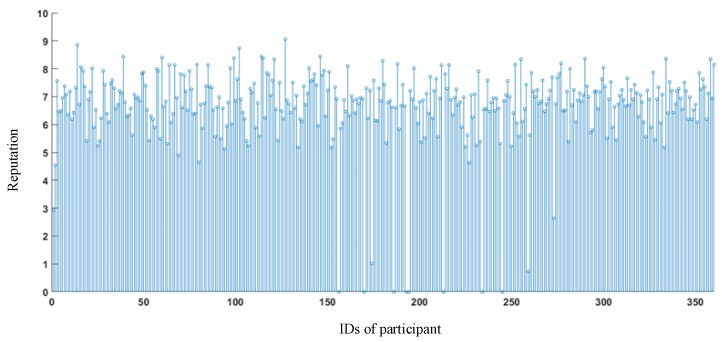
Reputation accumulation of participants.

Assume that the initial reputation of each participant is 5. UTH = 0.5, RTH = 3, and ζ = 1. The reputation accumulation of participants is illustrated in Figure 15. According to the statistics, for 1624 sensing tasks, 6037 malicious sensing data were filtered out and 562 person-times were punished. The payment cost of the sensing platform saved about $368.98.

## 7. Conclusions

In this paper, a staged incentive and punishment mechanism was explored for mobile crowd sensing. Our solution divides the whole incentive process into two stages: the recruiting stage and the sensing stage, and proposes a series of mechanisms to motivate potential participants to participate in the sensing task while balancing participants’ spatio-temporal data as well as the whole data utility. In addition, a reputation accumulation-based punishment mechanism was designed to introduce binding on the malicious participants. In this way, the payment cost of sensing task can be saved and resource waste of the sensing platform can be lowered effectively.

In future work, we plan to improve the incentive and punishment mechanism by introducing more influence factors into the existing incentive model, such as the type of sensing data, user participation, and so on.

## Figures and Tables

**Table 1:** Parameters and their meanings.

Parameter	Meaning
$T_{i}$	$i^{th}$ (time) period
$s_{j}$	$j^{th}$ task site
$U_{T_{i},s_{j}}$	the user set in the region $s_{j}$ during $T_{i}$
$R_{T_{i},s_{j}}$(*u*)	the number of check-ins in the region of $s_{j}$ during $T_{i}$ for user *u*
$task_{T_{i},s_{j}}$	the subtask in the region $s_{j}$ during $T_{i}$
En($U_{T_{i},s_{j}}$)	the information entropy in the region $s_{j}$ during $T_{i}$
$heat_{T_{i},s_{j}}$	the heat of $s_{j}$ during $T_{i}$
$c_{T_{i},s_{j}}$	the payment incentive coefficient of $task_{T_{i},s_{j}}$

**Table 2:** Crowd ratios at four task sites.

	Site 1	Site 2	Site 3	Site 4	Total Data Amount
PAIM	21.24%	19.86%	28.05%	30.84%	67,541
RAIM	16.38%	11.23%	28.52%	43.87%	60,531
SIM	24.20%	23.63%	24.46%	25.47%	70,069
Crowd ratio	16%	9%	30%	45%	1,074,427

## Abbreviations

The following abbreviations are used in this manuscript:

MCS	Mobile Crowd Sensing
LBSN	Location-Based Social Network
GPS	Global Positioning System
